# The Ins and Outs of miRNA-Mediated Gene Silencing during Neuronal Synaptic Plasticity

**DOI:** 10.3390/ncrna2010001

**Published:** 2016-01-11

**Authors:** Dipen Rajgor, Jonathan G. Hanley

**Affiliations:** Department of Biochemistry, University of Bristol, University Walk, Bristol BS8 1TD, UK; jon.hanley@bristol.ac.uk

**Keywords:** synaptic plasticity, synopase, miRNAs, ncRNAs, RNA binding proteins

## Abstract

Neuronal connections through specialized junctions, known as synapses, create circuits that underlie brain function. Synaptic plasticity, *i.e.*, structural and functional changes to synapses, occurs in response to neuronal activity and is a critical regulator of various nervous system functions, including long-term memory formation. The discovery of mRNAs, miRNAs, ncRNAs, ribosomes, translational repressors, and other RNA binding proteins in dendritic spines allows individual synapses to alter their synaptic strength rapidly through regulation of local protein synthesis in response to different physiological stimuli. In this review, we discuss our understanding of a number of miRNAs, ncRNAs, and RNA binding proteins that are emerging as important regulators of synaptic plasticity, which play a critical role in memory, learning, and diseases that arise when neuronal circuits are impaired.

## 1. Introduction

The mammalian brain is a complex structure, made up of millions of interlinked neuronal circuits that form through synaptic connections. A fascinating property of the brain is its ability to modify these neuronal circuits in response to various experiences such as learning new tasks, stress, and drug abuse [1,2]. Synaptic plasticity refers to modifications that occur to alter the strength of synaptic transmission at pre-existing synapses. Synaptic transmission can be either boosted or depressed by activity and these alterations can be short-term, lasting from milliseconds to hours, or long-term modifications that last for days to weeks or even longer [3,4,5,6]. Many mechanisms of synaptic plasticity have been described, demonstrating the complexity associated with changes in synaptic circuitry and transmission that constantly occur within the brain. Long-Term Potentiation (LTP) and Long-Term Depression (LTD) are two forms of long-lasting plasticity, which have been intensively investigated as models linked with memory and learning [3,4]. LTP is a form of plasticity resulting in a persistent enhancement of synaptic transmission and is widely believed to be important in memory formation, particularly in the hippocampal region of the brain which is involved in long-term memory formation [7,8,9,10]. LTD is a contrasting process, in which the efficacy of synaptic transmission is reduced [4].

**Figure 1:**
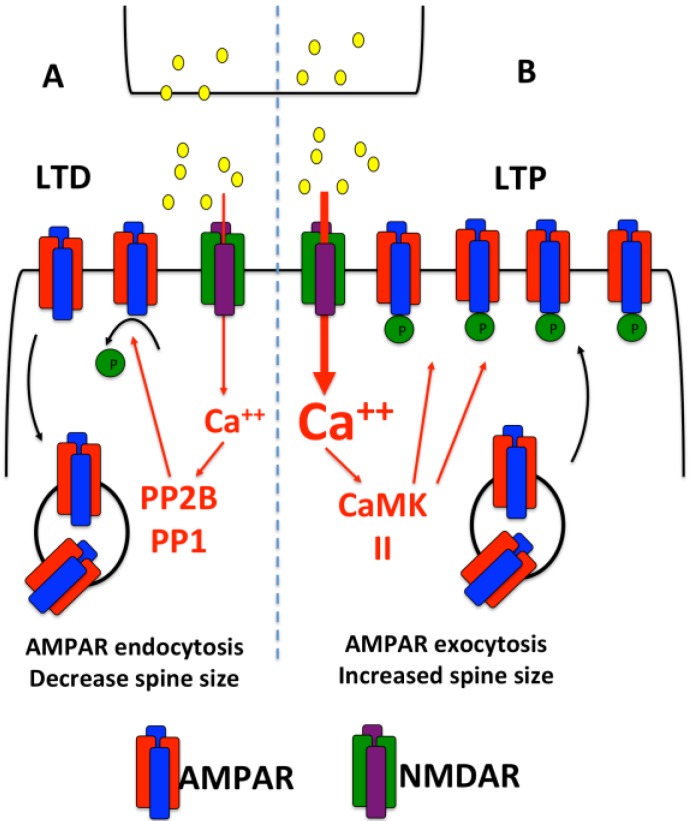
Long-Term Depression (LTD) and Long-Term Potentiation (LTP). (**A**) LTD occurs when a low rise in post-synaptic [Ca^++^] activates phosphatases that dephosphorylate AMPARs to reduce their sensitivity and promote their endocytosis; (**B**) LTP occurs when a larger rise in post-synaptic [Ca^++^] activates CaMKII, promotes AMPAR phosphorylation, and AMPAR insertion into the post-synaptic membrane.

α-Amino-3-hydroxy-5-methyl-4-isoxazolepropionic acid receptors (AMAPRs) are tetrameric assemblies of glutamate-gated ion channels present on the post-synaptic membrane. AMPARs are the major excitatory synaptic receptors in the brain and are tightly regulated to bring about changes in synaptic strength [11]. LTP and LTD induction causes activation of *N*-Methyl-d-aspartate receptors (NMDARs), which allows Ca^2+^ entry into the post-synapse. During LTP, a large influx of Ca^2+^ activates calcium/calmodulin-dependent protein kinase IIα (CaMKIIα). CaMKIIα activates downstream signaling cascades that increase spine size and the number and sensitivity of AMPAR inserted in the post-synaptic membrane [12,13,14] (Figure 1). In contrast, during LTD, a smaller influx of Ca^2+^ activates the protein phosphatase-1 (PP-1) signaling pathway, which dephosphorylates AMPARs and promotes their internalization to reduce post-synaptic surface receptors [15,16] (Figure 1). Furthermore, protein synthesis is reduced which results in shrinkage of the dendritic spine and in some cases even complete elimination of targeted spines [17].

Over the last decade, microRNAs (miRNAs) have emerged as key regulators of synaptic development and plasticity. miRNAs are an ever expanding class of small non-coding RNAs that function in post-transcriptional gene expression, and to date hundreds have been identified that are expressed in the brain [18,19,20]. miRNAs are transcribed by RNA polymerase II or III and are subsequently cleaved by the Drosha and DGCR8 containing microprocessor complex in the nucleus. The resulting pre-miRNA is exported to the cytosol and further processed by Dicer to an intermediate miRNA duplex. The leading miRNA strand is loaded into the miRNA-induced silencing complex (miRISC) and guided to target mRNAs to which it imperfectly pairs with sequences primarily in the 3′ untranslated regions (UTRs) of the mRNA. This interaction leads to translational repression of the target mRNAs and can be tightly regulated in neurons to control their rapid release and translation in response to specific stimuli to modify synaptic plasticity [21]. The promiscuous binding of miRNAs to target mRNAs allows a single class of miRNAs to repress multiple target transcripts involved in specific processes, which is an important feature for inducing synaptic plasticity by regulating local translation of dendritic transcripts during synaptic stimulation [22]. In this review, we discuss the importance of miRNAs and RNA binding proteins (RBPs) in synaptic plasticity and how they are able to rapidly induce synaptic changes by altering localized mRNA translation.

## 2. Dendritic Protein Synthesis

mRNA translation occurs in a compartmentalized manner within neurons to allow proteins to be synthesized locally to their sites of function [23]. Even within individual dendritic spines, synaptic proteins are translated at discrete sites to rapidly provide new proteins for synapses that require them [24]. Dendritic protein synthesis is regulated by a number of key factors, including miRNAs, RBPs and polyribosomes, which allow dendrites to undergo rapid synaptic plasticity in response to various synaptic stimuli.

Early studies identified polyribosomes within dendritic shafts and at the base of spines in neuronal cultures under basal conditions [25]. After intense synaptic activity, polyribosomes and translational initiation factors were observed to migrate into dendritic spines and localize at the postsynaptic density (PSD) of synapses [26,27,28,29]. PSDs are protein dense regions containing cytoplasmic proteins, membranous receptor proteins and receptor scaffold proteins underlying the postsynaptic membrane [30]. The re-localization of the protein synthesis machinery towards PSDs after intense synaptic activity implies neurons are capable of remodeling their synapses locally by translating new proteins.

Several studies have identified molecular mechanisms underlying mRNA transport from the cell body to dendritic spines. This process normally involves RNA species bound to RBPs as single mRNP complexes or present in clusters within RNA granules to be attached to microtubule-based motor proteins [31,32,33,34,35,36]. Originally, only a small number of mRNA species were thought to be transported to neuronal dendrites, but with advances in RNA sequencing techniques hundreds of different mRNAs have now been identified which are translated in an activity-dependent manner [22,37]. This includes transcripts for proteins extensively characterized to be involved in synaptic plasticity, for example, fragile X mental retardation protein (FMRP), AMPAR subunits GluR1/2, CaMKIIα, and LIM domain kinase 1 (LIMK1) [38,39,40,41].

Regulating protein synthesis in dendrites supports long-term changes in the growth and branching of spines, thus playing an important role in LTP, and in cognitive processes such as memory and learning [42,43,44,45]. For example, Fragile X syndrome (FXS) is the most common genetic disorder of intellectual disability worldwide. In FXS, the translational repressor protein, FMRP, is not expressed [46,47]. FMRP is a translational repressor which binds to ~4% of brain mRNAs and is particularly important in influencing synaptic plasticity. In FXS cells, neuronal protein synthesis is hyperactive under basal conditions, which disrupts dendritic morphology and synaptic strength [48,49,50,51]. Attempts to normalize protein synthesis levels in mouse models of FXS have proven successful in restoring behavioral deficits, suggesting that the translational machinery in neurons may represent novel therapeutic targets for memory and learning disorders [49,52,53].

## 3. Dendiritc miRNAs

**Figure 2:**
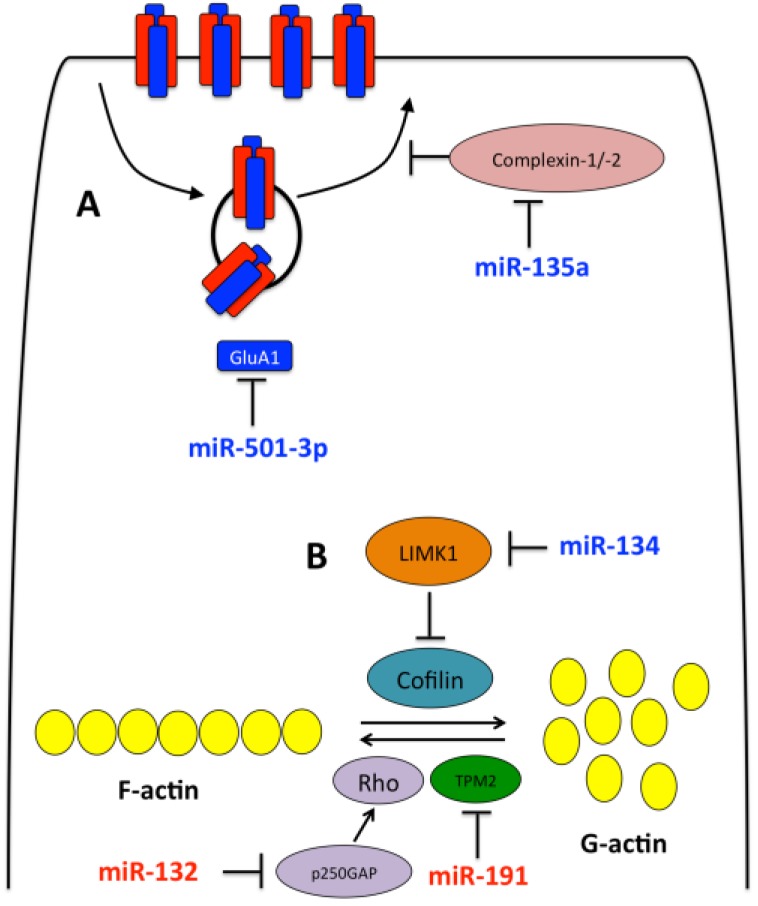
miRNAs involved in aclivity dependent AMPAR trafficking and dendritic structural plasticity. (**A**) miRNAs involved in NMDA-mediated AMAPR expression and trafficking. In response to NMDA, miR-501-3p is upregulated resulting in GluAl suppression and miR-135a is downregulated causing an increase in complexin-1/-2 levels and resulting in reduced AMAPR exocytosis; (**B**) miRNAs involved in dendritic structural plasticity miR-134 actrvity is enhanced in response to NMDA, resulting in downregulation of LIMK1 and activation of the actin depolymerizer, cofilin. Furthermore, expression of miR-132 and miR-191 increase, resulting in suppression of Rho signaling and Tropomodulin-2 (TPM2) respectively, thus repressing actin polymerization.

By exploiting miRNAs and RBPs involved in miRNA-mediated translational repression, neurons are well equipped to dynamically alter their proteomic profile in a localized manner. Here we discuss how some important miRNAs behave in an activity dependent manner to govern localized protein synthesis in dendrites and at synapses to influence synaptic plasticity (Figure 2, Table 1). 

**Table 1:** miRNAs involved in synaptic plasticity. miRNAs are important regulators of neuronal translation in the soma and within dendritic spines and synapses. This table summarizes key miRNAs identified within neuronal dendrites that have been identified to be major regulators in synaptic plasticity.

miRNA	Target	Refs.
miR-134	*LIMK1, Pum2*	[40,54]
miR-132	*p250GAP*	[55]
miR-125b	*NR2A*	[56]
miR-191	*Complexin-1, Complexin-2*	[37]
miR-135a	*TPM2*	[37]
miR-501-3p	*Gria1*	[57]
miR-138	*APT1*	[58]
miR-188	*NRP2*	[59]
miR-124	*Gria2*	[60]

### 3.1. miR-134

The actin cytoskeleton is essential for structurally re-modelling spines, and its dynamic rearrangement induced by synaptic activity are essential for this [61,62]. In response to LTD, filamentous actin (F-actin) in spines depolymerizes (Figure 2). Activation of the F-actin depolymerizing protein, cofilin, and inhibition of the actin polymerization complex, Arp2/3, are fundamental signalling cascades underlying actin reorganization for spine shrinkage in LTD [63,64].

miR-134 is a brain specific miRNA, embedded within the mammalian specific miRNA cluster (miR379-410). Schratt *et al.* demonstrated miR-134 is enriched in neuronal dendrites of hippocampal neurons, where it targets the *LIMK1* transcript [40]. Limk1 controls spine structure by regulating actin filament polymerization through binding to and inhibiting cofilin [65]. miR-134 mediated translational repression of Limk1 negatively regulates spine size and during cLTD enhanced miR-134 activity and a reduction in Limk1 has been observed, supporting its role in activity dependent spine re-modeling [66].

Further studies demonstrate miR-134, together with other miR379–410 members, is required for activity-dependent dendritogenesis in rat hippocampal neurons by fine-tuning Pumilio2 (Pum2) protein levels [54]. Pum2 is an RBP involved in translational repression and its regulation by miR-134 is key in activity-dependent plasticity [54]. Pum2-mediated miR134 repression illustrates a regulatory pathway that couples activity-dependent transcription of miRNA with miRNA-dependent translational control of gene expression in neuronal development, suggesting a possible cascade that might alter levels of multiple downstream effector genes.

*In vivo* experiments have provided a great tool for identifying the importance of miRNAs in memory formation and disease. Sirtuin-1 (SIRT1) is a nicotinamide adenine dinucleotide (NAD+)-dependent deacylase that has been linked with genome stability in neurons [67]. SIRT1 has been identified to modulate synaptic plasticity and memory formation by repressing miR-134 expression [68]. In the absence of SIRT1, increased activity of miR-134 caused down regulation of the transcription factor cAMP response element-binding protein (CREB), resulting in impaired synaptic plasticity. Additional *in vivo* studies have identified a functional role for miR-134 in specific periods of neuronal development and miR-134 has also been shown to play a role in neuroprotection and seizure suppression effects in mice [69].

### 3.2. miR-132

The neuronal enriched miR-132 was identified through a genome-wide screen as a CREB target [70]. Like many neuronal CREB targets, miR-132 is induced by neuronal activity and neurotrophins and plays a role in regulating neuronal morphology and excitability [71]. In cortical neuronal cultures, up regulation of miR-132 increases dendritic outgrowth in an activity-dependent manner by repressing GTPase-activating protein p250GAP translation, resulting in increased activation of the Rac1-PAK actin-remodeling pathway (Figure 2). In contrast, miR-132 inhibition attenuates neuronal outgrowth [55]. Furthermore, an additional study has demonstrated over expression of miR-132 in hippocampal neurons resulting in stubby and mushroom-shaped spines with an increase in protrusion size strengthening synaptic transmission [56]. miR-132 knockout mice additionally supported these findings, with their hippocampal neurons displaying reduced dendrite length, arborization, and spine density [72]. Together these studies support a role for miR-132 in regulating dendritic spine structures and synaptic transmission.

The tight regulation and fine-tuning of miR-132 expression is an important aspect of creating and controlling neuronal circuits. miR-132 was identified to be upregulated in the mouse hippocampus after presentation of spatial learning tasks [73]. Interestingly, miR-132 expression is downregulated in schizophrenia, with several miR-132 targets (DNMT3A, GATA2, and DPYSL3) displaying altered expression in tissue from adult schizophrenic subjects [74,75]. These experiments highlight the importance of buffering miR-132 levels over a fine concentration range for learning and memory formation and show the important roles miRNA fine tuning plays to regulate its downstream target genes.

Overall, studies on miR-134 and miR-132 demonstrate how complementary work *in vitro* and *in vivo* provides a powerful approach to dissecting the complex role miRNAs play during synaptic plasticity. These studies illustrate how miRNAs regulate multiple target genes at various stages in development to control both developmental and physiological plasticity.

### 3.3. Other miRNAs

miRNA regulation at the synapse is not only negative, for example, miR-125b mediates positive regulation of dendritic spine development [56]. miR-125b and several other miRNAs are associated with FMRP in mouse brain. miR-125b overexpression in hippocampal neurons results in longer, thinner dendrites and FMRP knockdown enhances the effect of miR-125b overexpression on spine morphology [56]. A mechanism has been proposed whereby FMRP phosphorylation provides a reversible switch in which Ago2 and miR-125a silence PSD-95 transcript. PSD-95 is key scaffold for positioning AMPARs in the post-synaptic membrane. During LTD, AMPARs are released from PSD-95, allowing them to laterally diffuse away from the synapse and be endocytosed [76]. Dephosphorylation of FMRP and the subsequent release of Ago2 from the PSD-95 mRNA, increases PSD-95 levels in the synapse and results in activation of mGluR signaling [77]. This switching mechanism could provide the means for temporal and spatial control of translation in response to post-synaptic receptor activation.

Recently, Hu *et al.* used next-generation deep sequencing to identify miRNAs differentially expressed in hippocampal neurons in response to chemically induced LTD (cLTD) [37]. They identified a substantial change in the miRNA transcriptome, with 34 upregulated and 36 downregulated miRNAs following cLTD. Enrichment analysis demonstrated many of these miRNAs induced changes in expression of target transcripts for proteins involved in synaptic transmission, actin-dependent processes, cytoskeletal binding proteins, and protein kinases and phosphotases, supporting previous work demonstrating that miRNAs play roles in the structural and functional plasticity of synapses [37]. Hu *et al.* studied the effects of miR-191 and miR-135a, which are downregulated and upregulated in response to cLTD, respectively. miR-135a represses tropomodulin-2, which is an actin filament-pointed end-capping protein that regulates the dynamics, length and amount of actin filaments [78]. They demonstrated that upregulation of miR-135a in response to cLTD is required to maintain reduced levels of F-actin beyond initial spine shrinkage and that this is important for long-lasting spine remodelling [37] (Figure 2). Hu *et al.* identified miR-191 to target complexin-1 and -2, which have a highly similar sequence. Both complexins are components of the SNARE complex involved in AMPAR exocytosis, and therefore important for inducing surface levels of AMAPRs for LTP [79,80]. Hu *et al.* identified that to maintain spine shrinkage during LTD, AMPAR exocytosis has to be inhibited by miR-135a translational repression of both complexins-1 and -2 [37] (Figure 2). In summary, this study demonstrated that expression changes of miR-191 and miR-135 after cLTD are required for maintenance but not induction of spine restructuring. Furthermore, actin depolymerization and AMPAR trafficking are regulated for extensive periods of time by miR-135a and miR-191 to support long-lasting spine plasticity [37].

In an additional study, the same authors performed miRNA pull-down experiments and computational prediction analysis to identify miR-501-3p as a target for the 3′ UTR of the transcript encoding AMPAR subunit GluA1 [57]. miR-501-3p was shown to increase locally in dendrites after NMDAR activation and this upregulation of miR-501-3p is required for NMDAR-dependent inhibition of GluA1 expression, long-lasting spine shrinkage, and elimination (Figure 2). Furthermore, the expression of miR-501-3p and GluA1 is inversely correlated during postnatal brain development. Together, these two studies by Hu *et al.* not only demonstrate the importance of miRNAs in activity-dependent local synthesis of dendritic AMPARs, but also their trafficking pathways.

## 4. Other Non-Coding RNAs (ncRNAs) in Synaptic Plasticity

As well as miRNAs, additional classes of ncRNAs such as Long non-coding RNA (lncRNA), piwi-RNAs, and circular RNA (circRNA) have been identified to be important in regulating protein synthesis in dendrites. In this section, we briefly describe how the long non-coding RNA *BC1*/*BC200* and circRNAs add an additional layer of complexity associated with protein synthesis in dendrites and synapses during plasticity.

### 4.1. Long Non-Coding RNAs

Long non-coding RNAs (lncRNAs) are non-protein coding transcripts that are longer than 200 base-pairs and, unlike miRNAs, have a low conservation between species [81,82]. lncRNAs are transcribed from various regions of the genome which include “gene desserts” between protein coding genes as well as regions which overlap two or more protein coding genes. Furthermore, they can be transcribed in both a sense and anti-sense manner allowing tens of thousands of lncRNAs to be transcribed [83,84]. The human genome contains ~25,000 protein coding genes and up to 20,000 lncRNAs are predicted to be expressed in the brain which suggests many lncRNAs are likely to have fundamental roles in brain function, including memory and learning [85]. Although not limited to, lncRNAs have been identified to function as “molecular sponges” for miRNAs and RBPs, while others provide platforms for assembling translational repression/activation mRNP complexes by binding to specific recognition motifs on mRNAs [82]. The function of lncRNAs is ever expanding, with new functional roles for different lncRNAs constantly emerging.

*BC1*/*BC200* was one of the first lncRNAs to be studied and is the only example to date of a lncRNA that regulates translation in neurons [86]. *BC1*/*BC200* regulates synaptogenesis and is present in neuronal dendrites where it interacts with FMRP and components of the translational machinery to control 48S complex formation and repress local translation in synapses [87,88]. *BC1*/*200* expression is reduced at synapses when neuronal activity is blocked and is therefore recognized as a putative plasticity gene that alters the proteome of synapses in response to neuronal activity [89]. Increased neuronal activity in a particular dendritic region would enhance local expression of *BC1*/*200* and negatively feedback on the rate of local translation. *BC1/BC200* knock-out mice have uncontrolled group I mGluR-stimulated synaptic protein synthesis, which results in excessive neuronal excitation causing convulsive seizures, anxiety, and behavioral defects [90,91,92]. Therefore translational control mediated by *BC1*/*200* is an essential neuronal plasticity mechanism that regulates neuronal activity and behavior.

### 4.2. Circular RNAs

Circular RNAs (circRNAs) are RNA species that form when the 5′ end of one exon and the 3′ end of another are covalently linked. Although the function of this class of ncRNA is not entirely clear, it is believed they play a role in post-transcriptional regulation [93,94]. For example, an elegant study by Memczak *et al.* demonstrated that human circRNA, antisense to the cerebellar degeneration-related protein 1 transcript (CDR1as), contains 63 binding sites for miR-7 [93]. CDR1 has a ~10 fold greater affinity to miR-7 than any other transcript and is able to act as a molecular “sponge” to sequester miR-7 suppressing other mRNAs. Interestingly, CDR1 is enriched in the brain, and when human CDR1as was expressed in zebrafish, it mimicked miR-7 knockdown by impairing midbrain development [93].

More recently, deep RNA profiling identified ~13,000 unique circRNA from five different tissues [95]. circRNAs were particularly enriched in the brain relative to other tissues and interestingly many were transcribed from genes encoding synaptic proteins. In some cases, circRNAs were more enriched in dendrites than their linear variants demonstrating expression of circRNAs are unlikely to be linked to expression of protein coding genes [95]. Furthermore, their expression was regulated throughout development and in response to neuronal activity, suggesting they are important for both synaptogenesis and plasticity. For example, when homeostatic plasticity was induced by treating hippocampal neurons with the GABAA receptor antagonist bicuculline, *circHomer1_a* (derived from the *Homer1* gene) levels were elevated ~5.5 fold whereas *Homer1a* mRNA levels only increased ~1.5 fold [95]. Homer1 plays a major role in the organization of PSDs and is important in mGluR signaling, suggesting GABAA signaling could influence mGluR signaling via a circRNA-mediated mechanism [96,97,98].

A key finding from this study suggested brain circRNAs are unlikely to serve as “molecular sponges” for miRNAs or RBPs [95]. This indicates brain circRNAs are likely to be involved in synaptic plasticity and synaptogenesis via a diverse set of new biological mechanisms that have yet to be identified. This study opens doors for future research where specific circRNAs can be overexpressed or depleted from neurons both *in vivo* and *in vitro* to identify their functions in brain development, disease, memory, and learning.

## 5. RNA Binding Proteins and Synaptic Plasticity

RNA granules are large assemblies of aggregated RNA-protein complexes that contain silent mRNAs in association with translational repressors, miRNAs, and/or specific RBPs. RNA granules are both highly heterogeneous in structure, protein, and RNA compositions, and highly dynamic as their dissolution correlates with translational activation [35]. mRNA Processing-bodies (P-bodies/PBs) are the best characterized RNA granules and are present in virtually all cell lines and model organisms studied. However, PBs in neuronal dendrites and synapses are different from PBs in non-neuronal cell lines, with several studies reporting that individual PBs within neurons can contain different protein compositions [99,100,101]. Interestingly, dendritic PBs respond to neuronal activity, for example, NMDAR stimulation induces the dissolution of a specific sub-type of PBs that contain the Decapping Co-activator Protein 1a (DCP1a), putatively releasing transcripts to allow their translation [99]. Recently, S-foci have been identified as a similar class of neuronal specific RNA granules that behave in a similar fashion [102]. S-foci contain the translational repressor Smaug1/Samd4a and associate to the post-synapse. Just like neuronal PBs, S-foci dissolve upon NMDAR activation. The *CamKII*a transcript was identified to be repressed in S-foci, and dissolution of S-foci correlates with increased translation of CamKIIα, suggesting RNA granules can carry transcripts for proteins important in synaptic plasticity, but more importantly play a role in regulating their translation [102,103].

Cytoskeletal remodeling in dendritic spines and at synapses is an important aspect of synaptic plasticity in LTP and LTD. β-actin mRNA and the Zip code Binding Protein 1 (ZBP1) have recently been shown to localize within dendritic granules under a masked state using single-molecule *in situ* hybridization [104]. Upon chemically-induced LTP, β-actin transcripts are released along with ribosomes to increase dendritic β-actin synthesis. Theoretically, numerous additional transcripts could go through similar masking/unmasking phases determined by neuronal activity and involving specific RBPs including FMRP, Pumilio, and several PB components.

Further studies still need to dissect the mechanisms that underlie RNA granule disassembly in response to neuronal activation. So far, the disassembly of S-foci upon NMDAR stimulation has been shown to occur in response to elevated localized Ca^2+^ levels and the activation of the PI3K/mTOR pathway [103]. However, the downstream mechanism other RNA granules use within dendrites to disassemble or modify their structures and compositions in response to synaptic stimulation has yet to be determined. These signaling cascades could potentially affect the way mRNPs within granules aggregate with direct consequences in granule organization and translational repression. RNA granules are associated with the cytoskeleton, and previous studies demonstrate the importance of the cytoskeleton in the dynamic properties of RNA granules [105]. Therefore, cytoskeletal remodeling that occurs during synaptic plasticity could participate in promoting granule dissolution or assembly, thus controlling translation or repression of certain proteins respectively.

## 6. Summary

Over the last decade, clear examples exist where ncRNAs and RBPs play a fundamental role in controlling synaptic plasticity. Certain miRNAs, such as miR-134 and -132, have been extensively studied and could provide good therapeutic targets for treating cognitive disorders. However, a large list of neuronal miRNAs, but particularly lncRNA and circRNAs, exist which have unidentified functions. Future work should focus on identifying their targets and elucidating how they are regulated in an activity dependent manner to regulate synaptic plasticity. Likewise, the function of multiple RBPs and their targets remain a mystery, particularly those localizing within granules. Additionally, the heterogeneous nature of neuronal granules may exist to allow different transcripts to be regulated in response to different stimuli and provide an additional layer of control in regulating synaptic plasticity.
